# TEAD1-dependent expression of the *FoxO3a *gene in mouse skeletal muscle

**DOI:** 10.1186/1471-2199-12-1

**Published:** 2011-01-07

**Authors:** Haifang Qiu, Fengli Wang, Chuxin Liu, Xuewen Xu, Bang Liu

**Affiliations:** 1 Key Laboratory of Agricultural Animal Genetics, Breeding and Reproduction of Ministry of Education, Huazhong Agricultural University, Wuhan 430070, China; 2 Weifang Bureau of Animal Husbandry, Weifang 261041, China

## Abstract

**Background:**

*TEAD1 *(TEA domain family member 1) is constitutively expressed in cardiac and skeletal muscles. It acts as a key molecule of muscle development, and trans-activates multiple target genes involved in cell proliferation and differentiation pathways. However, its target genes in skeletal muscles, regulatory mechanisms and networks are unknown.

**Results:**

In this paper, we have identified 136 target genes regulated directly by TEAD1 in skeletal muscle using integrated analyses of ChIP-on-chip. Most of the targets take part in the cell process, physiology process, biological regulation metabolism and development process. The targets also play an important role in MAPK, mTOR, T cell receptor, JAK-STAT, calcineurin and insulin signaling pathways. TEAD1 regulates *foxo3a *transcription through binding to the M-CAT element in *foxo3a *promoter, demonstrated with independent ChIP-PCR, EMSA and luciferase reporter system assay. In addition, results of over-expression and inhibition experiments suggest that *foxo3a *is positively regulated by TEAD1.

**Conclusions:**

Our present data suggests that TEAD1 plays an important role in the regulation of gene expression and different signaling pathways may co-operate with each other mediated by TEAD1. We have preliminarily concluded that TEAD1 may regulate *FoxO3a *expression through calcineurin/MEF2/NFAT and IGF-1/PI3K/AKT signaling pathways in skeletal muscles. These findings provide important clues for further analysis of the role of *FoxO3a *gene in the formation and transformation of skeletal muscle fiber types.

## Background

Myogenesis is a complex process regulated by a number of transcription factors, including myogenic determination factors Myf5 and MyoD, and differentiation factors myogenin, Myf4 and MEF2 [1]. Other factors, such as the TEA domain transcription factor family, also play vital roles in myogenesis. TEA domain proteins share a conserved DNA binding domain and govern developmental functions in a variety of animal and plant phyla [2,3]. TEAD1 is a member of the TEA domain family. Previous studies have indicated that *TEAD1 *is constitutively expressed in cardiac and skeletal muscles in pigs, mice and humans [4,5], and its disruption leads to heart defect and embryonic lethality in mice [6]. TEAD1 regulates the expression of many skeletal muscle-specific genes through binding to the M-CAT motif (TEAD1 protein binding site) in the promoters [7,8]. The transcriptional regulation of TEAD1 to muscle-specific genes is implemented by co-operating with numerous co-factors, including MEF2 [7], vestigial like 2 [9], vestigial like 4 [10], and so on.

Although mouse *TEAD1 *gene has been cloned and its DNA binding and trans-activation domains have been characterized, the target genes of TEAD1 are unknown. Considering the importance of TEAD1 to skeletal muscle development and the challenge of identifying direct gene targets of TEAD1 action, we have good reason to believe that chromatin immunoprecipitation combined with DNA microarray analysis (ChIP-on-chip) would be an effective approach to identify direct target genes of TEAD1. Moreover, we choose to focus on the adult skeletal muscle because it is a well-studied target of TEAD1 function in development.

Here, we identified 136 promoters significantly bound by TEAD1, and we found that 10 genes had more than 2 TEAD1 binding sites. We analyzed the functional categories and pathways of the target genes. Significantly, we found an important target gene, *FoxO3a*, which plays a critical role in muscle growth and development. Our data illustrate that TEAD1 is a mediator of skeletal muscle development.

## Results

### Identification of TEAD1-bound promoters by ChIP-on-chip analysis

With the aim of identifying the promoters bound by TEAD1, we performed ChIP-on-chip analysis. ChIP with a TEAD1 antibody was done using mouse skeletal muscle tissues. In two biological replicas, the promoter regions of 136 genes showed a reproducible signal (GEO accession number: GSE26107). All genes detected by the ChIP-on-chip assay are shown in Additional file 1, Table S1. There are 10 genes (*STOML1*, *F730014I05RIK*, *RBM34*, *A630050E13RIK*, *ZFP473*, *ZFP120*, *WDR73*, *TEF*, *SMARCAD1 *and *ARMCX1*), which have more than 2 putative TEAD1 binding sites.

**Figure 1:**
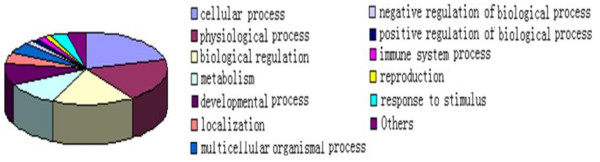
**Gene Ontology (GO) classifications of biological processes of TEAD1 target genes**. On the basis of the annotated genes that matched our unique tags, GO analysis was carried out using the DAVID tool.

To gain further insight into the biological importance of the target genes identified, we analyzed the functional categories of the annotated genes by examining their associated gene ontology. Most of the targets took part in the cell process, physiology process, biological regulation metabolism and development process (Figure 1). We then carried out pathway analysis examining the biological function of the targets, and have found that the target genes mainly take part in MAPK, mTOR, T cell receptor, JAK-STAT, calcineurin and insulin signaling pathways. These pathways are related to cell proliferation, differentiation, apoptosis, immunological regulation, growth and development.

### Validation of the FoxO3a gene with ChIP-PCR

**Figure 2:**
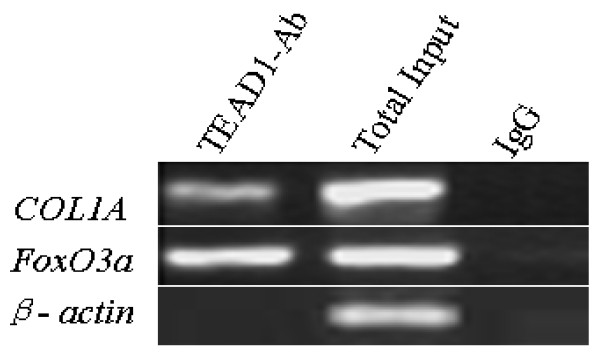
**Validation of the enrichment of the *FoxO3a *gene with ChIP-PCR using prepared TEAD1-IP, IgG-IP, and Input DNAs**.

In order to verify the importance of the *FoxO3a *gene found with ChIP-on-chip, we analyzed its enrichment using individual ChIP-PCR (primers used in Table 1). *β-actin *was used as a negative control, and *COL1A *(α1 chain of type I collagen) [11] was used as a positive control. Amplified TEAD1-IP or IgG-IP DNAs along with Input-DNAs from skeletal muscles of three mice were pooled, with equal quantities of DNA from each sample, and were then used as a template for PCR. Validation of the enrichment of the *FoxO3a *gene with ChIP-PCR is shown in Figure 2.

**Table 1:** Primers used in this paper.

Primer name	Primer sequnence
TEA-cdsF	5-CTAgctagcAACATGGAAAGGATGAGCGACT-3
TEA-cdsR	5-CCGctcgagTCAGTCCTTCACAAGCCTGTAGA-3
FOX-promoterF	5-GGATTGTGAAGGTGCGATCTG-3
FOX-promoterR	5-AGGTGTGTCACTGGACCCTCA-3
COL-F	5-GCCAGAGGTGCTGTCAC-3
COL-R	5-GGTGTGTCTGGCATGGCAG-3
FOX-forwardF	5-CCGctcgag GCAGTTGCTTTGTTCTGGTGAA-3
FOX-forwardR	5-CCCaagctt ACTCACAGGGAGCCTCAACCTA-3
FOX-reverseF	5-CCCaagctt GCAGTTGCTTTGTTCTGGTGAA-3
FOX-reverseR	5-CCGctcgagACTCACAGGGAGCCTCAACCTA-3
FOX-qpcrF	5-ATGTGACATGGAGTCCATCATCC-3
FOX-qpcrR	5-TGTCCACTTGCTGAGAGCAGAT-3
β-qpcrL	5-TGTACCCAGGCATTGCTGACA-3
β-qpcrR	5-GACTCATCGTACTCCTGCTTGCT-3
FOX-W	5-TGTGTGTTATTTTTGAGATGGAATGTGGGGCTGGCTGAACGGCT-3
FOX-T	5-TGTGTGTTATTTTTGAGATCTACCATGGGGCTGGCTGAACGGCT-3
FOX-WB	5-TGTGTGTTATTTTTGAGATGGAATGTGGGGCTGGCTGAACGGCT-3
siTEA-F1	5-GGUUCUUGCCAGAAGGAAATT-3
siTEA-R1	5-UUUCCUUCUGGCAAGAACCTG-3
siTEA-F2	5-GGGCGGACUUAAACUGCAATT-3
siTEA-R2	5-UUGCAGUUUAAGUCCGCCCAG-3
siTEA-F3	5-CACCUACCAGAGAAAUAUATT-3
siTEA-R3	5-UAUAUUUCUCUGGUAGGUGTT-3
siTEA-NF	5-UUCUCCGAACGUGUCACGUTT-3
siTEA-NR	5-ACGUGACACGUUCGGAGAATT-3

The restriction enzyme recognition sites are underlined

### Confirmation of TEAD1 binding to the FoxO3a promoter by EMSA

**Figure 3:**
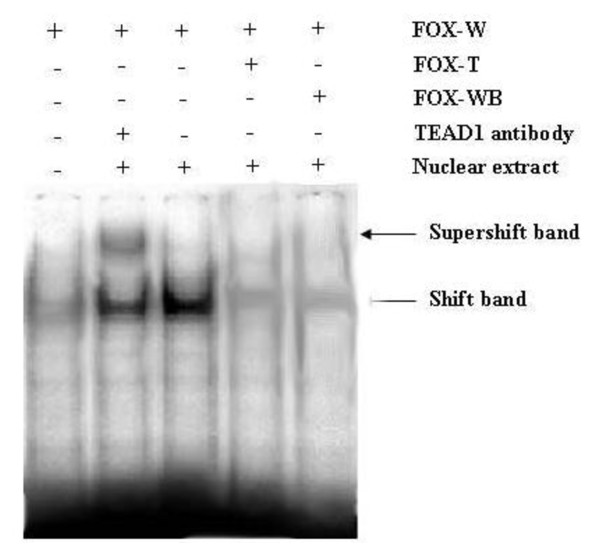
**EMSA analysis of TEAD1 binding site in the *FoxO3a *promoter**. Biotin-labeled oligonucleotide probes for the TEAD1 *FoxO3a *binding site were incubated with mouse skeletal muscle extracts. Competition experiments were performed using a 100-fold excess of mutant or unlabeled TEAD1 *FoxO3a *probes. Supershift assays were performed with anti-TEAD1 antibody. Arrows indicate the resulting bandshifts (FOX-W: normal probe; FOX-T: mutant probe; FOX-WB: unlabeled probe).

As described above, the TEAD1 binding site in the promoter region of the *FoxO3a *gene was confirmed with independent ChIP-PCR. We then employed EMSA assays to directly address whether TEAD1 binds *FoxO3a *in vivo. We synthesized specific oligonucleotides containing the TEAD1 element present in the *FoxO3a *promoter in EMSA experiments with nuclear extracts from mouse skeletal muscle tissues. As shown in Figure 3A, incubation of skeletal muscle nuclear extracts with biotin-labeled TEAD1-FoxO3a sequence produced a DNA-protein band shift. These DNA-protein complexes were determined to be specific to the TEAD1 site by successful competition assays using excess unlabeled TEAD1-FoxO3a and mutant TEAD1- FoxO3a oligonucleotides. To confirm the binding of TEAD1 to the TEAD1-FoxO3a sequence, these EMSA reactions were further incubated with anti-TEAD1 antibody. As shown in Figure 3B, the addition of this antibody resulted in a supershifted complex in addition to the DNA-protein band. These data confirm the presence of TEAD1 in the nuclear protein complex that binds the TEAD1 binding site of the FoxO3a promoter.

### The TEAD1 sequence is required for maximal transcriptional activity of the FoxO3a promoter in C2C12 cells

**Figure 4:**
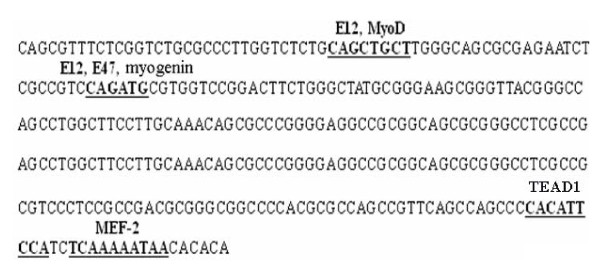
**The *TEAD1 *sequence is required for maximal transcriptional activity of the *FoxO3a *promoter in C2C12 cells, revealed by bioinformatics analysis**. The graphical representation shown is the sequence of a partial promoter of the *FoxO3a *gene. The cis-sequences are shown in boldface and underlined.

**Figure 5:**
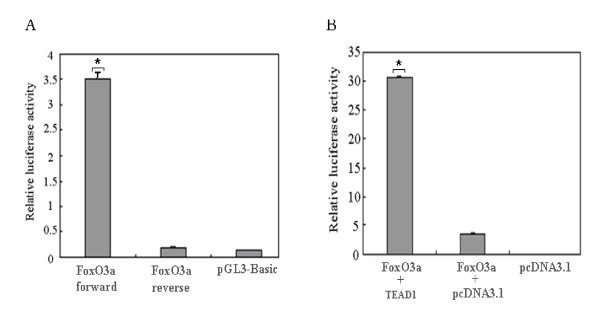
**Transient-transfection assays of *FoxO3a *promoter-luciferase reporters**. (A) A transient-transfection experiment in C2C12 cells was performed with a segment of *FoxO3a *cloned in either the forward or reverse upstream orientation of luciferase. The *y *axis of the graph represents the relative luciferase activity, with the transfected material shown on the *x *axis. The pGL3-Basic represents the luciferase vector lacking a promoter. (B) A transient-transfection assay was performed with the *FoxO3a *promoter-luciferase reporter transfected into C2C12 cells in the presence of a TEAD1 expression vector or the pCDNA3.1 vector as a control. The statistical significance of the differences between the *FoxO3a *forward and *FoxO3a *reverse, *FoxO3a *with TEAD1 and *FoxO3a *with pcDNA3.1 were analyzed by the Student's t-test, * P < 0.05.

Sequence analysis of a partial *FoxO3a *promoter revealed the presence of several consensus DNA sequences known to be required for the binding of muscle-specific transcription factors (Figure 4). Among them are E12, E47, MEF-2, MyoD, myogenin and TEAD1. To evaluate the functional importance of TEAD1 in mediating the transcriptional activity of the *FoxO3a *gene, the *FoxO3a *promoter was tested by transient transfection assays in C2C12 cells. We then cloned the *FoxO3a *promoter. If we were correct in assuming that the clone sequence represents the promoter of *FoxO3a *mRNA, then it should have promoter activity when inserted in the correct orientation with the transcription start-site upstream of the luciferase cDNA. The fragment was cloned in both orientations (forward and reverse) upstream of the luciferase cDNA and transfected into C2C12 cells, and promoter activity was measured. As shown in Figure 5A, only the fragment in forward orientation showed high promoter activity, whereas the one in reverse orientation did not. Importantly, the orientation showing promoter activity was the correct orientation of the fragment to drive *FoxO3a *mRNA transcription.

The ChIP-on-chip result shows that *FoxO3a *may be activated by TEAD1. To determine if TEAD1 binding influences the transcriptional activity of the *FoxO3a *promoter, we co-transfected the *FoxO3a *promoter-luciferase reporter construct with a TEAD1 expression construct, and pcDNA3.1 was applied as a negative control (Figure 5B). We found that, as expected, TEAD1 over-expression activated the *FoxO3a *promoter, and a 10-fold increase in reporter activity was observed. Thus, we can state that *FoxO3a *promoter was regulated by TEAD1. The exact mechanism by which TEAD1 regulates the *FoxO3a *promoter will be discussed further below (see Discussion).

### Effect of TEAD1 on FoxO3a transcription

**Figure 6:**
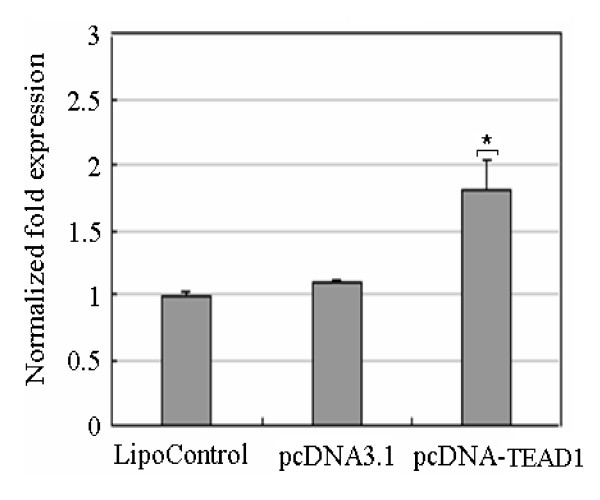
**Over-expression of TEAD1 experiment in C2C12 cells**. pcDNA3.1 vector or pcDNA-TEAD1 vector or lipofectamine 2000 control transiently transfected the C2C12 cells. *FoxO3a *expression was up-regulated about 2-fold compared to the control.

In order to characterize the effect of TEAD1 on *FoxO3a *transcription, firstly, we designed an over-expression experiment of TEAD1. We transiently transfected the C2C12 cells, which constitutively expressed high levels of *TEAD1*, measured using the pcDNA3.1 vector or pcDNA-TEAD1 vector or lipofectamine 2000 control. After 48 h, the cells were harvested and expression of *FoxO3a *was assessed by quantitative real-time PCR (Figure 6). The result showed that *FoxO3a *expression doubled as compared to the control.

**Figure 7:**
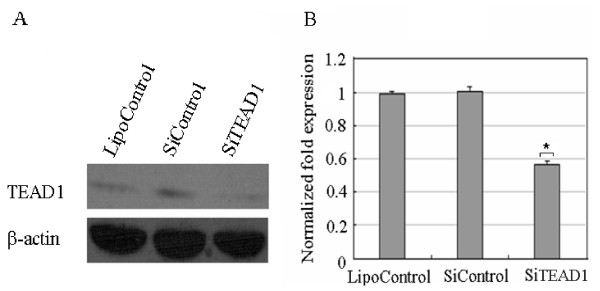
**Knock down of *TEAD1 *expression using siRNA**. (A) C2C12 cells were treated with siRNA against TEAD1 (SiTEAD1) or negative control (SiControl) or lipofectamine 2000 control (LipoControl) and western blot confirmed the suppression of TEAD1 in SiTEAD1 treated cells. (B) qRT-PCR analysis of *FoxO3a *using RNA extracted from SiTEAD1 or SiControl or LipoControl. *FoxO3a *expression was down-regulated about 50% compared to *β-actin*.

To further investigate the functional consequences of aberrant expression of TEAD1, we knocked down *TEAD1 *gene using the siRNA experiment. We first determined the optimal conditions for the introduction of the negative control siRNA with a FAM encoding fluorescent protein like GFP. By adjusting the conditions, we were ultimately able to express fluorescent proteins in ~70% of cells (data not shown). Under those optimized conditions, introduction of a mixture of three siRNA oligos targeting TEAD1 in C2C12 cells resulted in an 80% reduction in *TEAD1 *mRNA levels, as compared with cells transfected with control siRNA. After 48 h the cells were harvested and expression of *FoxO3a *was assessed by quantitative real-time PCR (Figure 7). *FoxO3a *expression was down-regulated by about 50%.

The over-expression and siRNA results were consistent, and they support the conclusion that TEAD1 is specifically bound to and regulates the expression of the *FoxO3a *gene. TEAD1 is also shown to be a mediator of skeletal muscle development.

## Discussion

ChIP-on-chip has emerged as a powerful tool for dissecting the complex network of regulatory interactions between transcription factors and their targets. It provides a new approach for examining direct transcription factor targets rapidly in an unbiased manner, which does not rely on previous characterization of a consensus sequence or a prior knowledge of gene expression patterns. In addition, we also used EMSA and the luciferase reporter system to verify the biological reliability of ChIP-on-chip data.

GO enrichment analysis shows that TEAD1 subtly regulates a large number of genes involved in the cellular process including cell proliferation, differentiation, growth, death and so on. In addition, some of the target genes of TEAD1 take part in MAPK, mTOR, T cell receptor, JAK-STAT, calcineurin and insulin signaling pathways. These pathways are related to cell proliferation, differentiation, apoptosis, immunological regulation, growth and development. These results add an additional layer of regulation to the effects of TEAD1 in promoting cell growth and development. Our results are in accordance with the reports suggesting that TEAD1 plays an important role in muscle development [12].

FoxO3a, a member of the Forkhead O (FOXO) family, has been shown to be involved in the regulation of skeletal and smooth muscle differentiation, proliferation and cell size regulation [13]. Mice lacking FoxO3a are apparently normal at birth, but develop cardiac hypertrophy and heart failure later in adult life [14,15]. *FoxO3a *is also a downstream target of the phosphatidylinositol-3 kinase (PI3K)/AKT pathway. It controls two decomposing systems of skeletal muscle proteins independently: ubiquitin/proteasome and autophagy/lysosome. When FoxO3a is activated, it causes muscle protein degradation and muscular atrophy through autophagy function. However, PI3K/AKT can inhibit the activity of FoxO3a, and hinder the atrophy process, thereby regulating the muscle atrophy [16,17]. Elia et al [18] reported that the activation state of the IGF-1 signal transduction cascade reciprocally regulates *miR-1 *expression through the FoxO3a transcription factor, and *miR-1 *expression was related to muscle cell growth and differentiation. All these results indicate that FoxO3a plays a critical role in muscle growth and development, thus, we have chosen this gene to validate as a potential target gene of TEAD1, and provide the basis for further function study.

TEAD1 can regulate transcription of the *foxo3a *gene through the binding to the M-CAT element, demonstrated with independent ChIP-PCR analysis, EMSA and luciferase reporter system assay. It is interesting to note that there is a miR-208b binding site in the antisense strand of *FoxO3a *(data not shown). MiR-208b is a muscle-specific miR, and plays redundant roles in the specification of muscle fiber identity by activating slow, and repressing fast myofiber gene programs [19]. Therefore, FoxO3a may play a key role in the process of the transformation of muscle fiber types. The muscle fiber type directly affects meat color, meat tenderness and intramuscular fat content. Therefore, future investigation of the effect of FoxO3a on regulating the transformation of muscle fiber types is essential.

MEF2 and NFAT bind in the promoter of *FoxO3a *(data not shown), which leads us to suppose that FoxO3a may take part in the calcineurin/MEF2/NFAT signaling pathway to promote the formation and transformation of skeletal muscle fiber types. At the same time, *FoxO3a *is also a downstream target of the PI3K/AKT signaling pathway [20]. Under normal conditions, PI3K and AKT are activated through insulin or insulin-like growth factor-1 (IGF-1) binding with their receptors, and then FoxO3a is phosphorylated and deactivated, so skeletal muscle can develop normally. When insulin resistance occurs or IGF-1 is lacking, this pathway is inhibited. FoxO3a is not phosphorylated, skeletal muscle protein is degraded, which causes muscular atrophy, and consequently, muscle fiber can not transform normally.

It was preliminarily concluded that TEAD1 regulates *FoxO3a *expression through calcineurin/MEF2/NFAT and IGF-1/PI3K/AKT signaling pathways in skeletal muscles. However, it is not known whether calcineurin/MEF2/NFAT functions as a downstream effector of the IGF-1/PI3K/AKT pathway or there is some other relationship. This question needs further study.

In summary, despite the stringency of the integrated analysis of ChIP and microarray data, novel TEAD1 candidate target genes were found and *FoxO3a *was shown as being directly regulated by TEAD1. Our data thus suggest that TEAD1 plays an important role in the regulation of gene expression and that different signaling pathways may co-operate with each other in a complex network of TEAD1 transcriptional regulation. These findings provide some important clues that will enable further analysis of the role of the *FoxO3a *gene in formation and transformation of skeletal muscle fiber types.

## Conclusions

Our current results provide the first insights into the target genes of TEAD1 transcription factor in skeletal muscles, and the regulatory mechanisms and networks involved. The over-expression and inhibition analysis suggest that *foxo3a *was positively regulated by TEAD1. In addition, we preliminarily concluded that TEAD1 may regulate *FoxO3a *expression through calcineurin/MEF2/NFAT and IGF-1/PI3K/AKT signaling pathways in skeletal muscles. These findings illustrate that TEAD1 is a mediator of skeletal muscle development, and also provide some important clues that will enable further analysis of the role of the *FoxO3a *gene in the formation and transformation of skeletal muscle fiber types.

## Methods

### Tissue sample collection and cell culture

All animal procedures were performed according to protocols approved by the Biological Studies Animal Care and Use Committee of Hubei Province, P. R. Skeletal muscle tissues were collected from adult Kunming mice, then frozen in liquid nitrogen, and stored at -80°C. C2C12 cells were cultured in DMEM/high glucose supplemented with 20% FBS, 100 units/ml penicillin and 100 μg/ml streptomycin and maintained at 37°C in 5% CO2.

### Plasmid construction

**Figure 8:**
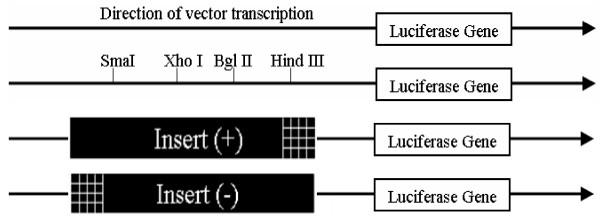
**Schematic representation of the pGL3-Basic vector and the relative positions of the restriction sites used in these experiments**. The arrow represents the direction of transcription of the vector. The (+) and (-) orientations are defined as the positioning of the insert with respect to the luciferase gene in the same or reversed direction as it occurs with respect to the *FoxO3a *coding sequence.

The mouse *TEAD1 *and *FoxO3a *gene sequences were obtained from GenBank (NM_009346 and NC_000076) and were employed to designed primers (Table 1). For this purpose, we utilized LA Taq (TaKaRa, Japan), and cloned the amplicons into the pMD18-T (Takara) vector for subsequent manipulation. The coding region of the *TEAD1 *gene was excised from pMD18-T (Takara) by NheI-XhoI digestion, and blunt-end ligated into the pcDNA3.1 to construct the expression vector pcDNA-TEAD1. Using the same method, the promoter of *FoxO3a *containing the TEAD1 binding site (5'-CATTCCT-3') was insert into the XhoI-HindIII and HindIII-XhoI sites of the pGL3-Basic vector (Promega, Madison, WI) to generate forward (+) and reverse (-) luciferase reporter constructs (Figure 8). The reporter vector pGL3-Basic does not contain any eukaryotic promoter or enhancer elements. The forward vector was defined as having the 1.865-kb insert ligated into pGL3-Basic with the same 5'-3' orientation relative to the reporter gene as the native sequence in the genomic clone relative to the *FoxO3a *gene.

### Luciferase reporter assay

The C2C12 cells were seeded into 96-well plates at an initial density of 60-80% and cultured overnight to ensure adhesion and spreading. Co-transfections were then performed using 0.5 μl of Lipofectamine 2000 reagent (Invitrogen, Carlsbad, CA, USA) with 200 ng of the appropriate firefly luciferase plasmid DNA, and 20 ng of pRL-TK plasmid DNA (Promega) as an internal control. For co-transfection analyses, the levels of reporter plasmids were kept constant, but 100 ng of pcDNA-TEAD1 vector was added. The pGL3-Control vector (Promega) was used as a positive control. The transfection medium was removed and replaced with a growth medium after 4 to 6 hours. Firefly and Renilla luciferase activities were measured at 48 hours post-transfection using the Dual-Glo Luciferase assay system (Promega). The transfection efficiencies in each case were normalized using the Renilla luciferase activity levels and each construct was tested in triplicate in a minimum of three independent experiments.

### Validation of gene expression by qRT-PCR

qRT-PCR was performed using the SYBR Premix Ex Taq Kit (TaKaRa) with cycling conditions consisting of an initial 5 min at 95°C, followed by 35 cycles comprising 15 sec at 95°C, 20 sec at 61°C, 20 sec at 72°C, and fluorescence acquisition at 83°C for 1 sec. PCR (primers used in Table 1) was then performed in triplicate and the gene expression levels were quantified relative to the expression of the *β-actin *gene using the Gene Expression Macro software (Bio-Rad, Hercules, Calif., USA), employing an optimized comparative Ct (ΔΔCt) value method. Dissociation curves were generated to ensure that a single amplicon had been produced.

### Western blot analysis

Western blot analyses were performed as described [21]. Total protein concentration in the samples was determined by the Bradford assay (Bio-Rad). Antibody against TEAD1 was mouse monoclonal antibody from BD (BD Transduction Laboratories, San Diego, CA). Anti-β-actin antibody used as a control was a mouse monoclonal antibody from Santa Cruz (Santa Cruz Biotechnology, Santa Cruz, CA). The images were quantified using image J software from NIH.

### siRNA and transfection

For TEAD1 knockdown studies, cells plated in 6-well plates were transfected using Lipofectamine 2000 reagent. siRNA was designed by Invitrogen. Three siRNA and control sequences targeting TEAD1 were present (Table 1). Cells were harvested after 48 hours and the RNA was isolated using an RNAEasy extraction kit (Qiagen, Valencia, CA, USA).

### Electrophoretic mobility shift assay (EMSA)

Nuclear extracts were prepared from mouse skeletal muscles using the NE-PER Nuclear and Cytoplasmic Extraction Reagents (Pierce, Rockford, IL). A double-stranded oligonucleotide probe containing the putative TEAD1 binding site was labeled at the 5'-end with biotin by commercial company (Table 1). Incubation for the DNA binding reaction was performed using the Lightshift Chemiluminescent EMSA Kit (Pierce). For competition experiments, a 100-fold excess of mutant or unlabeled double-stranded oligonucleotide probe (Table 1) was added to the reaction. For supershift experiments, 3 μg of antibody against TEAD1 (BD) was added to the reaction mixture and incubated with the nuclear extracts at room temperature for 20 min before the addition of the probe. Protein-DNA complexes were electrophoresed on a 4% native polyacrylamide gel in buffer containing 45 mmol/L Tris, 45 mmol/L boric acid, and 1 mmol/L EDTA, pH 8.3, at 4°C. The samples were then transferred to a positively charged nylon membrane. Finally, a charge-coupled device camera (FluorChem, Astec) was used to detect chemiluminescence on the membrane.

### Chromatin immunoprecipitation

**Figure 9:**
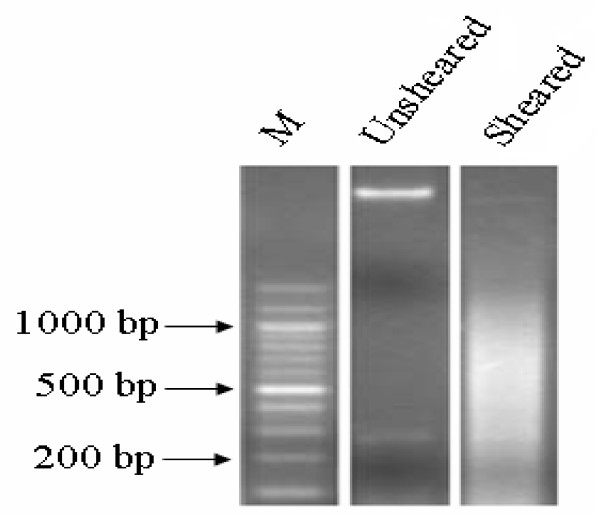
**Yield and size of DNA precipitated with anti-TEAD1 antibody from mouse skeletal muscle**.

ChIP assays were performed on mouse skeletal muscles. Briefly, 1 g of tissue was fixed with formaldehyde, neutralized with glycine and rinsed with cold phosphate-buffered saline. After lysis, samples were sonicated to an average DNA length of 1000 bp (Figure 9). Following chromatin sonication, the lysates were precipitated overnight using 5 μg of mouse anti-TEAD1 monoclonal antibody (BD) and anti-IgG antibody (BD) as a negative control. Two independent ChIP experiments were performed for each antibody. Protein A-agarose beads were added to purify immune complexes. Cross-linking was reversed by heating the samples overnight at 65°C. RNA was degraded with RNase A for 4 h at 65°C, and proteins were degraded by proteinase K treatment for 2 h at 45°C. The DNA was further purified. The ChIP-captured DNA of samples, IgG DNA and the total input DNA consisting of genomic DNA prepared from control cross-linked tissues were prepared for amplification.

### Promoter array hybridization, data analysis, statistics and criteria of significance

To obtain the target genes of TEAD1, the mouse promoter array, M8K (Aviva Systems Biology, San Diego, CA), containing 8000 40-mer oligonucleotide probes targeting gene promoters was used. The DNA samples were amplified and labeled by Aviva's ChIP-GLAS system. Briefly, ChIP, IgG, and input DNAs were biotinylated and annealed to the M8K-Oligo-Mix. The DNA samples were then amplified by ligation mediated PCR with T3 and T7 based primers (25 cycles), labeled with Atto 550 or 647, and hybridized onto the chip. Amplification, labeling, hybridization, and washing were carried out according to the manufacturer's instruction (Aviva, AK-0524). Slides were scanned with the DNA Microarray Scanner BA (Agilent Technologies, Santa Clara, CA, USA) and intensities were extracted with the Feature Extraction Software Version 9.1 (Agilent Technologies, Santa Clara, CA, USA). Raw data were read into the R statistical computation environment for preprocessing and data analysis [22]. Local background intensities were subtracted from raw signals and negative values were replaced with small positive ones. Signals were then normalized using a variance stabilization method described in [23,24] and implemented in the vsn Bioconductor package [25]. Functional annotation of the target genes, including classification and pathway analysis, was carried out using the DAVID tool [26-28]. Promoter sequences were analyzed for the presence of putative transcription factor binding sites using the TESS (Transcription Element Search System) web tool available from the Computational Biology and Informatics Laboratory at the University of Pennsylvania.

## Authors' contributions

HFQ performed the experiments and drafted the manuscript. FLW participated in the design of the study and assisted in statistical analysis. CXL helped in vector construction experiments. XWX supplied technical expertise and assisted in editing the manuscript. BL designed, coordinated and helped to draft the manuscript. All the authors have read and approved the final manuscript.

## Supplementary Material

Additional file 1 **The target genes of TEAD1 obtained by ChIP-on-chip**. The table presents the target gene ID, gene symbol, gene description, ratio of TEAD1/IgG and number of TEAD1 binding sites. Click here for file
